# Pathway Processor 2.0: a web resource for pathway-based analysis of high-throughput data

**DOI:** 10.1093/bioinformatics/btt292

**Published:** 2013-06-05

**Authors:** Luca Beltrame, Luca Bianco, Paolo Fontana, Duccio Cavalieri

**Affiliations:** ^1^Translational Genomics Unit, Department of Oncology, Istituto di Ricerche Farmacologiche ‘Mario Negri’ IRCCS, Via La Masa 19, 20159 Milano, Italy and ^2^Research and Innovation Center, Edmund Mach Foundation, Via E Mach 1, 8010 S. Michele all’Adige, Italy

## Abstract

**Summary:** Pathway Processor 2.0 is a web application designed to analyze high-throughput datasets, including but not limited to microarray and next-generation sequencing, using a pathway centric logic. In addition to well-established methods such as the Fisher’s test and impact analysis, Pathway Processor 2.0 offers innovative methods that convert gene expression into pathway expression, leading to the identification of differentially regulated pathways in a dataset of choice.

**Availability and implementation:** Pathway Processor 2.0 is available as a web service at http://compbiotoolbox.fmach.it/pathwayProcessor/. Sample datasets to test the functionality can be used directly from the application.

**Contact:** duccio.cavalieri@fmach.it

**Supplementary information:** Supplementary data are available at *Bioinformatics* online.

## 1 INTRODUCTION

The advance of high-throughput methods, particularly because of the advent of next-generation sequencing (NGS) provides an unprecedented amount of data from a single experiment. Although analysis method handling such data has considerably improved, comparing and especially integrating results between different platforms and analysis systems is still a challenge.

For these reasons, there is a need for newer methods able to handle this type of data effectively and in a robust manner.

In recent years, pathway-based approaches have emerged as a way to compare, integrate and interpret results from different ‘omics’ experiments (Beltrame *et al.*, 2009; Manoli *et al.*, 2006). In particular, pathway-based approaches can be useful methods to investigate complex phenotypes (Rizzetto *et al.*, 2010, 2012). The main advantage of these methods is a greater ease of interpretation and increased comparability among different experiments, methodologies and platforms. Methods using pathways have evolved considerably as well, starting from the classical Fisher’s test (Grosu *et al.*, 2002), over more complex systems such as Gene Set Enrichment Analysis (Subramanian *et al.*, 2005) and impact analysis (Khatri *et al.*, 2012). In addition, newer methods have been proposed, which target RNA-seq datasets specifically in addition to microarrays: one of these is the Gene Set Variation Analysis (GSVA) (Haenzelmann *et al.*, 2013), which shifts the focus from gene expression to pathway expression through the generation of enrichment scores. Although methods like the Fisher’s test are commonly available in bioinformatics software, more advanced algorithms are often just part of customized software pipelines, and as such are not potentially useful to biologists. Also, a part of this software was developed with microarrays in mind, and while most algorithms are platform agnostic, often adapting them to newer technologies requires a considerable effort. Here, we describe Pathway Processor 2.0, a substantial upgrade over the original Pathway Processor introduced in 2002 (Grosu *et al.*, 2002). Developed as a web-based software, Pathway Processor 2.0 aims at using pathway-based approaches on omics data to extract meaningful and biologically sound information to support the biological hypothesis being tested. To do so, it offers well-established statistical methods in addition to a new method to calculate differential pathway expression between two user-supplied phenotypes.

## 2 IMPLEMENTATION

Pathway Processor 2.0 was implemented as a web-based service, using PHP for the graphical interface and for the analysis relies on a back-end written using R and Python. The back-end carries out the pre-processing, analysis and generation of final results, whereas the front-end handles the selection of analysis type, the input parameters and the display of input and results. Special care was taken to make the analysis back-end independent of the input platform and in fact Pathway Processor 2.0 supports any type of high-throughput data (microarray, RNAseq and proteomics).

Pathway Processor 2.0 can analyze high-throughput data using four different methods, divided by the type of the input data and the possibility of using custom pathways (Table 1; Supplementary Data). The Fisher’s test is implemented as in the original Pathway Processor, but with some important improvements: in particular, multiple species are supported (currently human, mouse, rat, yeast and fruit fly) as long as the supplied identifiers for Differentially Expressed Genes (DEGs) and ‘gene universe’ (the whole list of genes on microarray chip or the complete list of genes of the organism under investigation) are in the correct format (Entrez Gene ID, RefSeq or gene symbols). Furthermore, in addition to the built-in KEGG pathway set, any custom gene set can be analyzed, by uploading an archive file containing the pathways (or gene sets) of interest. Visualization of genes over significant KEGG pathways is also possible (Supplementary Data). The user has to be aware that the statistical significance of the Fisher’s Exact Test is affected by the size and the connectivity of the gene set tested. Therefore, the results from this test have to be considered as a rapid and user-friendly way to discover the biological processes to be further investigated and verified experimentally.

**Table 1.** Analysis methods available for Pathway Processor 2.0

Method name	Input type	Custom gene sets
Fisher’s test	DEGs + gene universe	Yes
Impact analysis	DEGs + gene universe	No
Gene ontology	DEGs + gene universe	No
GSVA	Normalized data	Yes

The impact analysis method (Draghici *et al.*, 2007; Tarca *et al.*, 2009), which allows to determine activation or inhibition of pathways depending on the alteration of the genes involved and the topology of the pathways themselves, is implemented in Pathway Processor 2.0 using the improved version present in the ‘graphite' R package (Sales *et al.*, 2012), which provides a fully updated pathway model for KEGG, Reactome and the Pathway Interaction Database. Currently, impact analysis is limited to data from *Homo sapiens* only.

Gene Ontology analysis is implemented through the R ‘topGO’ package, using a weighted algorithm over the whole Gene Ontology tree to select the significant affected nodes (Alexa *et al.*, 2006).

In all cases, analysis parameters can be adjusted to fine-tune the results (Supplementary Data), and all data files produced can be downloaded from the web server for further analysis. Multiple test correction procedures are used to control the rate of false positives (Supplementary Data).

The third analysis offered by Pathway Processor 2.0 is the application of the recently developed GSVA, coupled with linear models for differential expression analysis. This method, given a set of pathways and normalized gene expression data, allows the transformation of the data into pathway enrichment scores (a measure of the state of each pathway), generating a *pathway expression matrix*. This matrix is then used for a comparison of two user-supplied phenotypes of interest using moderated *t*-tests as implemented in the R package ‘limma’ (Smyth, 2005). The final result is a list of Differentially Regulated Pathways (DRPs; Supplementary Data) that can then be used to interpret data with a pathway-based view, providing more information in elucidating complex phenotypes. The GSVA matrix can also be downloaded, enabling its use in other downstream applications.

All the analyses can be run on the web server after uploading the required files, without the need for any local installation of additional software or analysis tools. The software’s performance is in line with existing solutions (Supplementary Data).

## 3 CONCLUSIONS

Pathway Processor 2.0 is a useful tool to analyze ‘omics’ datasets, regardless of the platform that produced them, usable with both microarays and next-generation sequencing data. The web-based interface provides a one-stop shop to well-tested bioinformatic algorithms, and the new methods included in this software enable interpretation of the data with a true pathway-based view, allowing for deeper insight into complex biological problems.

## Supplementary Material

Supplementary Data
