# Supplementary material for: Pathway Processor 2.0: a web resource for pathway-based analysis of high-throughput data
Source: Bioinformatics. 2013 Jun 5;29(14):1825–6. doi: 10.1093/bioinformatics/btt292 (PMC3702260; doi:10.1093/bioinformatics/btt292)
Supplement: Supplementary Data [file supp_29_14_1825__index.html]

Pathway Processor 2.0: a Web resource for pathway-based analysis of high throughput data — Pathway Processor 2.0: a web resource for pathway-based analysis of high-throughput data — Pathway Processor 2.0: a web resource for pathway-based analysis of high-throughput data — Supplementary Data 

# Pathway Processor 2.0: a web resource for pathway-based analysis of high-throughput data

## 

files

**Files in this Data Supplement:**

- Supplementary Data - pdf file
