# Supplementary material for: Pathway Processor 2.0: a web resource for pathway-based analysis of high-throughput data
Source: Bioinformatics. 2013 Jun 5;29(14):1825–6. doi: 10.1093/bioinformatics/btt292 (PMC3702260; doi:10.1093/bioinformatics/btt292)
Supplement: Supplementary Data [file supp_btt292_Supplementary_data.pdf]

Supplementary data

Supplementary Text 1. Analysis types, supported inputs and identifiers.

Supplementary Text 2. Multiple test correction support.

Supplementary Text 3. Benchmarks to evaluate performance.

Supplementary Figure 1. The main interface of Pathway Processor 2.0 when running Fisher's test or impact analysis.

Supplementary Figure 2. Example results from a Fisher's test run. DEGs, differentially expressed genes.

Supplementary Figure 3. Example results from an impact analysis run. pSize, number of genes in the pathway; tA, amount of change observed and which direction; pGFdr, FDR-corrected p-value from the test.

Supplementary Figure 4. Example results from a Gene Ontology run.

Supplementary Figure 5. Display of a significant pathway from an impact analysis result showing the up- and down-regulated genes. Green indicates down-regulated genes, while red highlights up-regulated genes.

Supplementary Figure 6. The main interface of Pathway Processor 2.0 when using GSVA.

Supplementary Figure 7. Selection of case and control samples from a GSVA run.

Supplementary Figure 8. Example results from a GSVA run showing Differentially Regulated Pathways (DRPs). LogFC, log2 fold change; t, moderated t-test statistic; B, B-statistic.

Supplementary Text 1. Analysis types, supported inputs and identifiers.

Depending on the type of analysis, different combinations of identifiers and data types are possible in Pathway Processor 2.0:

Analysis type	Species	Identifiers	Data type	Pathway sets
Fisher's Test	Human, mouse, rat, yeast, fruit fly	Entrez ID, RefSeq, gene symbol	DEGs and gene universe	KEGG, custom sets
Impact analysis	Human only	Entrez ID, RefSeq, gene symbol	DEGs and gene universe	KEGG, Reactome, NCI-PID
Gene Ontology	Human, mouse, rat, yeast, fruit fly	Entrez ID, RefSeq, gene symbol	DEGs and gene universe	Biological Process, Cellular Component, Molecular Function
GSVA	Human, mouse, rat, yeast, fruit fly	Entrez ID, RefSeq, gene symbol	Normalized data*	KEGG, custom sets

*, In the case of microarray data, log2-transformed absolute expression values; in case of RNAseq data, counts or RPKM.

Supplementary Text 2. Multiple test correction support.

For Fisher's Test, p-values are corrected following the procedure employed by Benjamini and Hochberg (2005), using the "statsmodels" Python package (McKinney, Perktold and Seabold, 2011). Impact analysis uses the built-in FDR correction provided by the R "SPIA" package (same method as above), while the GSVA test uses the same correction as directly employed in the R "limma" package (Smyth GK, 2005).

Supplementary Text 3: Benchmarks to evaluate performance.

In order to profile Pathway Processor's use of GSVA, a GSVA test run was performed on a set of 6 samples (Illumina dataset E-MTAB-751 from Array Express) with 48802 features using the Pathway Processor engine and R (which served as reference). The test pathway set was the entire Broad C2 collection of 3272 gene sets. For Pathway Processor the initial overhead of setup was included in the benchmark.

The test was repeated 10 items. Both processes were run in parallel over 8 processors using GSVA's built-in parallelism. Annotation of gene sets to the dataset was done through GSVA.

The fastest time for R was 69 seconds, while for Pathway Processor the fastest time was 66 seconds. The difference was negligible, and it was as expected, because Pathway Processor uses the R GSVA package to do the calculation, called from the Python backend.

Pathway Processor had some additional overhead for each run when loading pathway sets during normal operation, as they needed conversion to R objects (approximately 7 seconds in the best case for a 850 element pathway set from Reactome), but the difference with R was made even by a much faster I/O and data manipulation than native R thanks to the use of the "pandas" library

(http://wesmckinney.com/blog/?p=543).

ISTITUTO AGRARIO DI SAN MICHELE ALL'ADIGE

Fondazione Edmund Mach

Pathway Processor 2.0 ?

PATHWAY PROCESSOR 2.0: Fisher / GO / Impact Analysis

Select Diff. Expr. Genes file ?	E-MTAB-751-DEG.txt
Select Platform Genes file ?	E-MTAB-751.txt
Choose ID type ?	ENTREZID ▾
Choose the type of Analysis ?	Fisher Test ▾
Choose the Species ?	Homo Sapiens ▾
P-value cutoff ?	0.05
Fold change type ?	Fold Change ▾
Pathways in PWF (.zip) ?	Scegli file Nessun file selezionato

Submit

Copyright © 2012 Fondazione Edmund Mach. All Rights Reserved.

Istituto Agrario di San Michele all'Adige

Fondazione Edmund Mach

Pathway Processor 2.0

Please click on pathway links to see KEGG's graphical representation

Show/Hide Parameters

New Analysis

Export Results

Pathway	DEGs in pathway	No. of genes	p-value	Adjusted p-value
Rheumatoid arthritis	16	92	0	0
Cytokine-cytokine receptor interaction	33	265	0	0
Toll-like receptor signaling pathway	16	102	0	0
NOD-like receptor signaling pathway	11	59	0	0
Chagas disease (American trypanosomiasis)	13	104	1e-9	4.52e-8
Amoebiasis	11	106	1.22e-7	0.00000459533333333
Hematopoietic cell lineage	10	88	1.77e-7	0.00000571457142857
Leishmaniasis	9	73	2.18e-7	0.0000061585
Osteoclast differentiation	11	128	8.4e-7	0.000018984
Jak-STAT signaling pathway	12	155	8.36e-7	0.000018984
Chemokine signaling pathway	12	189	0.000005792	0.000109082666667
Prion diseases	6	35	0.000005666	0.000109082666667
Type I diabetes mellitus	6	45	0.000020184	0.000350891076923
Malaria	6	51	0.000034447	0.000556073
Measles	9	134	0.00004847	0.000730281333333
African trypanosomiasis	5	35	0.000075933	0.001072553625
T cell receptor signaling pathway	8	108	0.000084747	0.00112663658824
Cytosolic DNA-sensing pathway	6	62	0.000096307	0.00120918788889
Graft-versus-host disease	5	43	0.000151978	0.00180773831579

Copyright © 2012 Fondazione Edmund Mach. All Rights Reserved.

Istituto Agrario di San Michele all'Adige
Fondazione Edmund Mach

Pathway Processor 2.0

Please click on pathway links to see KEGG's graphical representation

Show/Hide Parameters

New Analysis

Export Results

Pathway	Annotated	Significant	Expected	pvalue	Adjusted_pvalue
inflammatory response	439	46	7.09	6.1e-17	2.65594e-13
response to lipopolysaccharide	198	28	3.2	1.4e-13	3.0478e-10
immune response	942	62	15.22	9e-10	0.0000013062
positive regulation of interferon-gamma production	28	8	0.45	9.6e-9	0.0000104496
cell-cell signaling	998	28	16.13	1.3e-7	0.000113204
chronic inflammatory response	17	6	0.27	1.8e-7	0.00013062
response to hypoxia	208	15	3.36	3.1e-7	0.00019282
positive regulation of interleukin-6 production	30	7	0.48	3.9e-7	0.0002122575
defense response to Gram-positive bacterium	32	7	0.52	6.2e-7	0.000299942222222
negative regulation of viral genome replication	22	6	0.36	0.000001	0.0004354
lipopolysaccharide-mediated signaling pathway	35	7	0.57	0.0000012	0.000474981818182
anti-apoptosis	257	20	4.15	0.0000019	0.000689383333333
positive regulation of smooth muscle cell proliferation	38	7	0.61	0.0000022	0.000736830769231
positive regulation of osteoclast differentiation	15	5	0.24	0.0000028	0.0008708
positive regulation of inflammatory response	65	12	1.05	0.0000038	0.00110301333333
positive regulation of nitric oxide biosynthetic process	29	6	0.47	0.0000058	0.00154808888889
response to glucocorticoid stimulus	130	12	2.1	0.0000062	0.00154808888889
signal transduction	3819	125	61.71	0.0000064	0.00154808888889
toll-like receptor 4 signaling pathway	84	9	1.36	0.0000082	0.00187909473684

Copyright © 2012 Fondazione Edmund Mach. All Rights Reserved.

ISTITUTO AGRARIO DI SAN MICHELE ALL'ADIGE

Fondazione Edmund Mach

Pathway Processor 2.0

Please click on pathway links to see KEGG's graphical representation

Show/Hide Parameters

New Analysis

Export Results

Pathway	pSize	tA	pGFdr	Status
Cytokine-cytokine receptor interaction	249	36.1253167011	2.81653056603e-20	Activated
Toll-like receptor signaling pathway	98	-14.9612820418	2.57581354184e-10	Inhibited
Rheumatoid arthritis	85	-1.1405091725	3.36512202455e-8	Inhibited
Osteoclast differentiation	122	36.9515281346	7.04430757208e-8	Activated
NOD-like receptor signaling pathway	58	-7.68306261543	2.81901489438e-7	Inhibited
Chagas disease (American trypanosomiasis)	99	6.76388013422	6.30504018418e-7	Activated
MAPK signaling pathway	255	26.5603690518	0.00000207493367748	Activated
Amoebiasis	101	2.99759768047	0.0000423999798175	Activated
Jak-STAT signaling pathway	148	-1.19082855118	0.0000423999798175	Inhibited
Chemokine signaling pathway	178	20.5772381263	0.0000489223128642	Activated
Leishmaniasis	65	-2.24696325465	0.000120460971468	Inhibited
Measles	124	-2.94800868278	0.00137250230862	Inhibited
Prion diseases	34	-3.99560635562	0.00139441593379	Inhibited
Malaria	46	0	0.00172786034951	Inhibited
Apoptosis	86	25.0100966128	0.00172786034951	Activated
African trypanosomiasis	33	1.49599388412	0.00208993490513	Activated
T cell receptor signaling pathway	104	-2.37231990242	0.00208993490513	Inhibited
Graft-versus-host disease	38	-1.23582372364	0.00404925322054	Inhibited
Amyotrophic lateral sclerosis (ALS)	51	16.5465702224	0.00654458843345	Activated

Copyright © 2012 Fondazione Edmund Mach. All Rights Reserved.

Toll-like receptor signaling pathway - Homo sapiens (human)

Help

[Pathway menu | Organism menu | Pathway entry | Download KGML | Show description | User data mapping]

Homo sapiens (human) ▾ Go 100% ▾

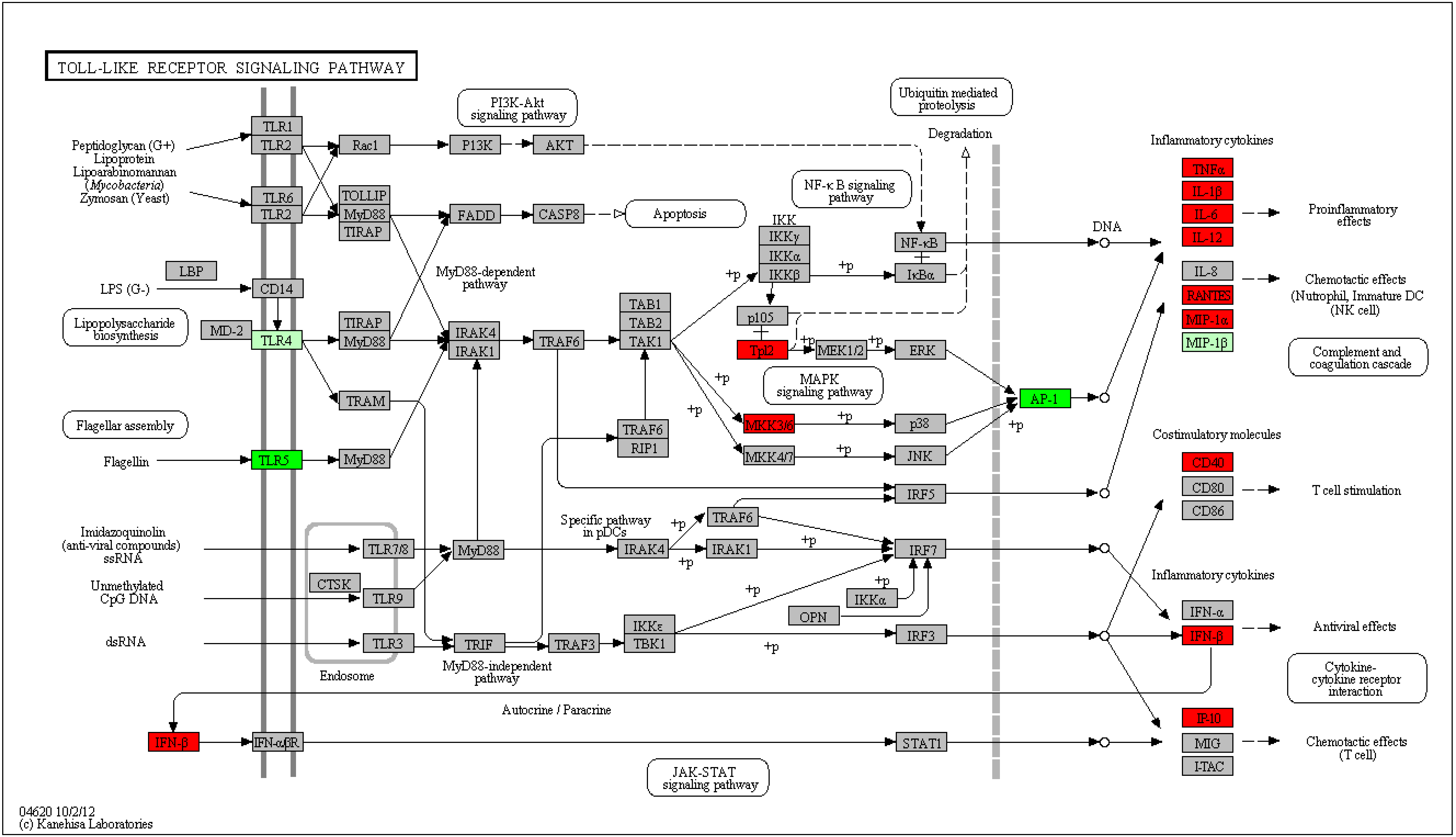

ISTITUTO AGRARIO DI SAN MICHELE ALL'ADIGE
Fondazione Edmund Mach

Pathway Processor 2.0

Please click on pathway links to see KEGG's graphical representation

PATHWAY PROCESSOR 2.0: GSVA

Select Normalized Input ?	GSE40419.txt
Choose ID type ?	ENTREZID ▼
Choose the type of Data ?	RNA-Seq ▼
Choose the Species ?	Homo Sapiens ▼
P-value cutoff ?	0.05
Pathways in PWF (.zip) ?	Scegli file Nessun file selezionato

Submit

Copyright © 2012 Fondazione Edmund Mach. All Rights Reserved.

ISTITUTO AGRARIO DI SAN MICHELE ALL'ADIGE

Fondazione Edmund Mach

Pathway Processor 2.0 ?

Please Specify at lease one Case and one Control Samples ?

Sample	None	Case	Control
BM_CD34_R000030	○	●	○
BM_01000	○	●	○
BM_02300	○	●	○
BM_02303	○	○	●
BM_02347	○	○	●
BM_02417	○	○	●
BM_02496	○	○	●
BM_02619	●	○	○
BM_02680	●	○	○
BM_02879	●	○	○
BM_030065	●	○	○
BM_CD34_R000121	●	○	○
BM_CD34_TBO3488	●	○	○
BM_CD34_TBO2249	●	○	○
BM_CD34_TBO2523	●	○	○
BM_CD34_TBO2841	●	○	○

Copyright © 2012 Fondazione Edmund Mach. All Rights Reserved.

ISTITUTO AGRARIO DI SAN MICHELE ALL'ADIGE

Fondazione Edmund Mach

Pathway Processor 2.0

Please click on pathway links to see KEGG's graphical representation

Show/Hide Parameters

New Analysis | Export GSVA Matrix | Export Results

Pathway	logFC	t	P val	Adj. P val	B
African trypanosomiasis	0.7678884	4.4459563	0.0000547	0.0019715	1.5341806
Linoleic acid metabolism	0.7004568	4.0564755	0.0001904	0.0036486	0.3429148
Cytokine-cytokine receptor interaction	0.614048	3.5658288	0.0008581	0.0101489	-1.0822384
Caffeine metabolism	0.6084181	3.2982965	0.0018793	0.0180102	-1.8161673
Neuroactive ligand-receptor interaction	0.5860291	3.3728109	0.0015151	0.0158401	-1.615149
Arrhythmogenic right ventricular cardiomyopathy (ARVC)	0.5646402	3.2226422	0.0023331	0.0206388	-2.017438
Complement and coagulation cascades	0.5393983	3.1202841	0.0031136	0.023101	-2.2850593
Dorso-ventral axis formation	0.5360507	2.9223638	0.0053656	0.030734	-2.7864226
alpha-Linolenic acid metabolism	0.5232818	2.8800268	0.0060132	0.030734	-2.890785
ECM-receptor interaction	0.5204179	2.9185418	0.0054213	0.030734	-2.7958869
RIG-I-like receptor signaling pathway	0.5175654	2.9331267	0.0052116	0.030734	-2.7597258
Steroid hormone biosynthesis	0.5123155	2.9669566	0.0047542	0.030734	-2.6753789
Retinol metabolism	0.5109351	2.9706926	0.004706	0.030734	-2.6660242
Glycosphingolipid biosynthesis - lacto and neolacto series	0.5033617	2.9005062	0.0056913	0.030734	-2.840433
Maturity onset diabetes of the young	0.5023335	2.7590279	0.0082864	0.0381175	-3.183188
Jak-STAT signaling pathway	0.4916984	2.8322208	0.0068315	0.0334306	-3.0073614
Malaria	0.4813741	2.7911205	0.0076164	0.0364951	-3.1064941
Hedgehog signaling pathway	0.4762149	2.6914838	0.009878	0.0436912	-3.3425276
Pyrimidine metabolism	-0.4862893	-2.7205493	0.0091614	0.0413163	-3.2743085

Copyright © 2012 Fondazione Edmund Mach. All Rights Reserved.
